# Standardized Terminology and Potential Taxonomic Utility for Hadrosaurid Skin Impressions: A Case Study for *Saurolophus* from Canada and Mongolia

**DOI:** 10.1371/journal.pone.0031295

**Published:** 2012-02-03

**Authors:** Phil R. Bell

**Affiliations:** Department of Biological Sciences, University of Alberta, Edmonton, Alberta, Canada; Raymond M. Alf Museum of Paleontology, United States of America

## Abstract

The characterization of palaeospecies typically relies on hard-tissue anatomy, such as bones or teeth that is more readily fossilized than soft parts. Among dinosaurs, skin impressions are commonly associated with partial and complete hadrosaurid skeletons, and consist of non-imbricating tubercles or scales. Skin impressions from various parts of the body of two species of the hadrosaurine *Saurolophus* (*S. angustirostris* from Mongolia and *S. osborni* from Canada) are described from multiple specimens. These species, recently validated on osteological grounds, can be differentiated based solely on soft-tissue anatomy, namely scale shape and pattern. This study demonstrates for the first time the applicability of soft-tissue (i.e., scale impressions) as a means to differentiate species within the Dinosauria. Differences are most spectacular in the tail, where *S. angustirostris* is differentiated by the presence of vertical bands of morphologically distinct scales, a grid-like arrangement of circular feature-scales, and tabular scales along the dorsal midline. Preliminary results indicate scale architecture remained consistent throughout ontogeny in *S. angustirostris*. These results support previous assertions that hadrosaurid scale architecture has a positive phylogenetic signal. As such, future taxonomic descriptions should include, where possible, the standardized description of skin impressions including the position and orientation of these impressions on the body.

## Introduction

The characterization of extant and Recent vertebrate species typically relies on features of the soft-tissue (feathers, scales, hair), which rapidly degrade after death. Subsequently, and in direct contrast, most palaeospecies are typified by hard-parts (bones and teeth) that are more prone to fossilization. Among dinosaurs, preserved vestiges of the integument (including scales, feathers, and ossified dermal structures [e.g. scutes]) are known from most major groups, including theropods, sauropods, thyreophorans, and cerapodans [1]–[16], yet few attempts have been made to characterize species based on these structures [16]–[18]. Among the best represented in terms of preserved soft-tissue is Hadrosauridae, for which skin impressions are known from virtually all parts of the body [2], [16], [19], [20]. Despite the relatively rich fossil record of hadrosaurid integument, only a small number of studies have alluded to the potential use of these structures in taxonomy [16], [19].

In 1947, the Russian Palaeontological Expedition to Mongolia's Gobi Desert, led by I. A. Efremov, discovered a bonebed of the giant hadrosaurine, *Saurolophus angustirostris* Rozhdestvensky 1952 in the Nemegt Formation. The bonebed became known as the “Dragon's Tomb”, from which numerous articulated skeletons, many with skin impressions, were recovered. The reliability of this site as a producer of well-preserved, articulated dinosaur skeletons has unfortunately made it a favorite target for poachers, and untold numbers of *Saurolophus* specimens have been destroyed in the process. Regardless, the Dragon's Tomb continues to yield spectacular skin impressions, and many undescribed specimens have been deposited in museums around the world.

Although not as extensive, skin impressions were found with the holotype (AMNH 5220) and paratype (AMNH 5221) of *Saurolophus osborni* Brown 1912 from the upper Horseshoe Canyon Formation in southern Alberta, Canada. These specimens, collected by B. Brown and P. Kaisen in 1911, included skin impressions from the jaw, pelvis, pes, and tail but were never mentioned in the original descriptions [21], [22] of that species and have subsequently evaded study. A third specimen (AMNH 5271), also collected by Brown, preserves extensive skin impressions along the tail. This specimen lacks the skull but is attributed to that taxon on account of the relatively low neural spines on the dorsal vertebrae, which differentiate it from *Hypacrosaurus altispinus*, the only other hadrosaur known from that stratigraphic level.

Species of *Saurolophus* are osteologically very similar, sharing—in addition to their typical hadrosaurian beauplan—a solid, rod-like cranial crest composed of the nasals, frontals, and prefrontals [23], [24]. Their overall similarity has led some authors to question the validity of *S. angustirostris* [25]; however, recent studies have validated both species as distinct taxa [23], [24]. Whereas previous studies have commented on the taxonomic utility of hadrosaur scale morphology at the generic level [16], [19], the preserved integument on the two species of *Saurolophus* provides a unique opportunity to investigate this theory at the species level. The purpose of this paper is threefold: 1) to provide a standardized terminology for scale descriptions; 2) to describe the integument of *S. osborni* and *S. angustirostris*; and 3) to provide a comparison of the soft tissues between these two species and elucidate for the first time areas of soft tissue not previously observed among hadrosaurs.

### Suggested terminology for scale morphology

In an attempt to simplify and standardize terminology used for describing scale morphology and general arrangement, a terminology is proposed herein. This system is not intended to be comprehensive for Dinosauria; however, certain terms and definitions will and do apply to groups outside of Hadrosauridae although they will not be elaborated on here. The proposed system is intended as a starting point to be built upon and refined by other workers as new specimens and scale morphologies are found.

**Figure 1:**
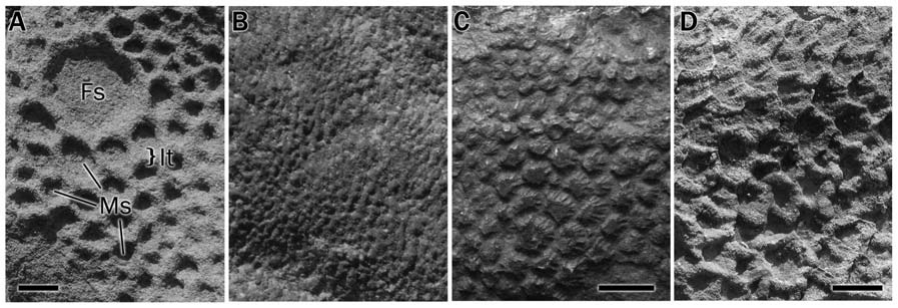
Hadrosaur scale morphology. (A) Polygonal basement-scales (Ms) with a shield feature-scale (fs). Interstitial tissue (It) occurs between scales (*Saurolophus angustirostris*); B. Pebbles (*S. angustirostris*); (C) Radially-ornamented, irregular basement-scales (*Edmontosaurus annectens* ROM 801); (D) Imbricated shell basement-scales (*S. angustirostris*). Scale bars = 1 cm.

Herein, the terms ‘basement’ or ‘basement-scales’ are used to describe the scales that form the major part of the integumentary surface. Collections of similar-sized basement-scales in a mosaic-like arrangement were described as ‘cluster areas’ by Osborn ([2]: 42) and this terminology is followed here. Basement-scales form the background pattern onto which larger and sporadically arranged scales are often imposed (Fig. 1A). These larger scales are frequently (but not invariably) of different morphology to the basement-scales and are referred to as ‘feature-scales’. One or more type of feature scale may be present in a single anatomical region (e.g., hind limb) on a single individual but may be absent altogether. Feature scales present along the dorsal midline above the neural spines as in *Gryposaurus notabilis* (ROM 764), and cf. *Edmontosaurus* sp., (MOR V 007; [5]) are termed ‘midline feature-scales’. ‘Interstitial-tissue’ refers to the integument between the scales and presumably afforded the skin its ability to flex and fold.

Several types of scale morphologies are recognized in the integument of *Saurolophus* (Table 1) and are defined as follows:

**Table 1:** Scale morphology and descriptive terminology.

	Size	Type	Ornamentation
**Basement-scales**	1–10 mm	Polygonal	Smooth; striated
		Pebbles	Smooth
		Irregular	Smooth; corrugated
		Shells	Corrugated
**Feature-scales**	>7 mm	Polygonal	Smooth
		Irregular	Smooth; corrugated
		Shield	Smooth; corrugated; striated
		Multi-pointed shield	Smooth; corrugated

Polygonal scales may range from four- to six-sided. Although three-sided and greater-than-six sides have not been observed, they would also fall into this category. Sizes range from ∼2 mm to more than 10 mm in greatest dimension and may be symmetrical or asymmetrical. Polygonal scales may form basement-scales, feature-scales, or both.

Pebbly scales, or pebbles, are always small and form a basement of closely-packed, rounded nodes (Fig. 1B). They are apparently the smallest of the scales, typically measuring only about 1 mm in diameter.

Shell scales are asymmetrical, and somewhat trapezoidal in shape (Fig. 1C). Shells form matrices that are often imbricated to some degree. The most critical feature are the anteroposteriorly oriented corrugations that give each scale the overall appearance of some bivalvular mollusc shells from which the name is derived.

Scales without obvious geometrical sides are termed irregular. Often, the circumference is wavy or indistinct. These are of variable size (>2 mm in diameter) and may form basement- or feature-scales. Irregular scales can also have radial striae (corrugations), giving them a “wrinkled” appearance, which add to their irregular outline (Fig. 1D).

Shield scales are circular or ovoid and interspersed among the surrounding scales as feature-scales (Fig. 1A). Consequently, they are notably larger than the surrounding basement-scales, ranging from 7 mm to several centimetres in diameter and have been referred to as “limpet-like” by some authors [19], [26]. Shields are typically flat or domed, and their surfaces may be smooth or corrugated. A variation of shield scales includes a series of triangular points around the circumference of the central shield and is referred to as a multi-pointed shield. The individual points tend to intervene between adjacent basement-scales or may lie in a wide area of interstitial tissue.

Anatomical directions such as dorsal or ventral reference the scales' relationship relative to the axial midline of the animal as they appear in most two-dimensional fossils. In the case of a three-dimensional animal, the ventral edge of a scale underhanging the belly where the outer (superficial) surface faced the ground refers to the edge that may have been facing somewhat medially in life.

### Institutional Abbreviations

**AMNH**, American Museum of Natural History, New York, New York, USA; **CMN**, Canadian Museum of Nature, Ottawa, Ontario, Canada; **JRF**, Judith River Foundation/Judith River Dinosaur Institute, Malta, Montana, USA; **MOR**, Museum of the Rockies. Bozeman, Montana, USA; **NMMNH**, New Mexico Museum of Natural History and Science, Albuquerque, New Mexico, USA; **PIN**, Palaeontologiceski Institut, Academii Nauk, Moscow, Russia; **ROM**, Royal Ontario Museum, Toronto, Ontario, Canada; **TMP**, Royal Tyrrell Museum, Drumheller, Alberta, Canada; **UALVP**, University of Alberta, Edmonton, Alberta, Canada; **UW**, University of Wyoming Geological Museum, Wyoming, USA; **ZPAL**, Institute of Palaeobiology of the Polish Academy of Sciences, Warsaw, Poland.

## Methods

Due to the immense quantity of material available at the Dragon's Tomb, only a limited number of specimens were collected. Collection was further hindered due to the extreme hardness of the entombing rock. Where possible, small hand samples were collected that had been previously unearthed and broken by poachers. In order to obtain voucher specimens that represent different body parts, two methods of molding were also employed in the field. Initial attempts used quick-setting modeling clay, which was applied directly on to the rock surface. The clay was pressed onto the impressions and removed before it had time to set (less than five minutes). Cyanoacrylate and/or acryloid was then applied to strengthen the molds. Despite good results, shrinkage and the fragility of the final product rendered this method less than ideal. A second method using liquid two-part silicone (Dragon's Skin™, Smooth-On Inc., Easton, Philadephia, USA) provided excellent results. Dragon's Skin™ was poured directly onto the skin impressions and allowed to cure overnight before being removed. Anti-adhesive sprays were not used, because the silicone peels did not penetrate the heavily indurated sandstone and thus separated easily from the rock surface. All observations and measurements were taken from the original specimens and supplemented by the casts. Measurements were taken using a measuring tape and/or calipers from the original specimens to avoid discrepancies on the molds from shrinkage due to drying, which was apparent in the larger clay molds. For all other specimens (i.e., not from the Dragon's Tomb locality), only the actual specimens were used.

No specific permits were required for the described field studies; however, permission was granted by the Mongolian Palaeontological Centre (Ulaan Baatar) to the expedition leads (Y.-N. Lee and P. J. Currie [Korea-Mongolia International Dinosaur Project]; M. Ryan and D. Evans [Cleveland Museum of Natural History and Royal Ontario Museum with Nomadic Expeditions]) on behalf of the respective expeditions for access to the field site.

## Results

### Skull and mandible

**Figure 2:**
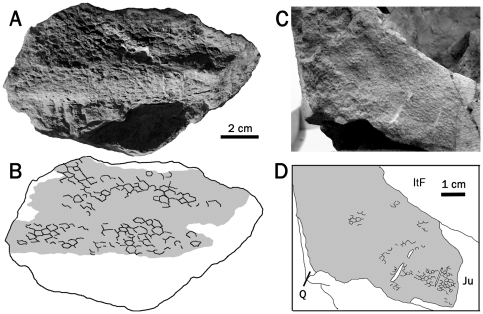
Cranial integument of *Saurolophus*. (A, B) *Saurolophus osborni* (AMNH 5220) right dentary. (C, D) Juvenile *S. angustirostris* (ZPAL-MgD-1/159). Itf, infratemporal fenestra; Ju, jugal; Q, quadrate. Grey areas denote regions of skin impressions.

Skin from the face of *Saurolophus osborni* is known from a single fragment from the right dentary of AMNH 5220. It measures 14 cm anteroposteriorly and 5.5 cm dorsoventrally (Fig. 2). Basement-sales on the posteroventral surface are anteroposteriorly longer than high (5×3 mm) with no apparent variation. They form rounded, vaguely diamond-shaped, raised tubercles. More dorsally situated scales are diamond or hexagonal in shape, anteroposteriorly longer than high, ranging from 5–6 mm long and 3–4 mm high. Scales appear to increase in size anteriorly and feature-scales are absent.

At least two patches of faintly preserved skin are present on a juvenile specimen of *Saurolophus angustirostris* (ZPAL-MgD-I/159). Skin is preserved on the right quadrate-quadratojugal-jugal contact (40×50 mm) and the left infratemporal fenestra (30×40 mm). Scales comprise small (1 mm), closely-packed, hexagonal-to-subcircular pebbles. These are uniformly distributed with no apparent pattern or variation.

### Axial region

**Figure 3:**
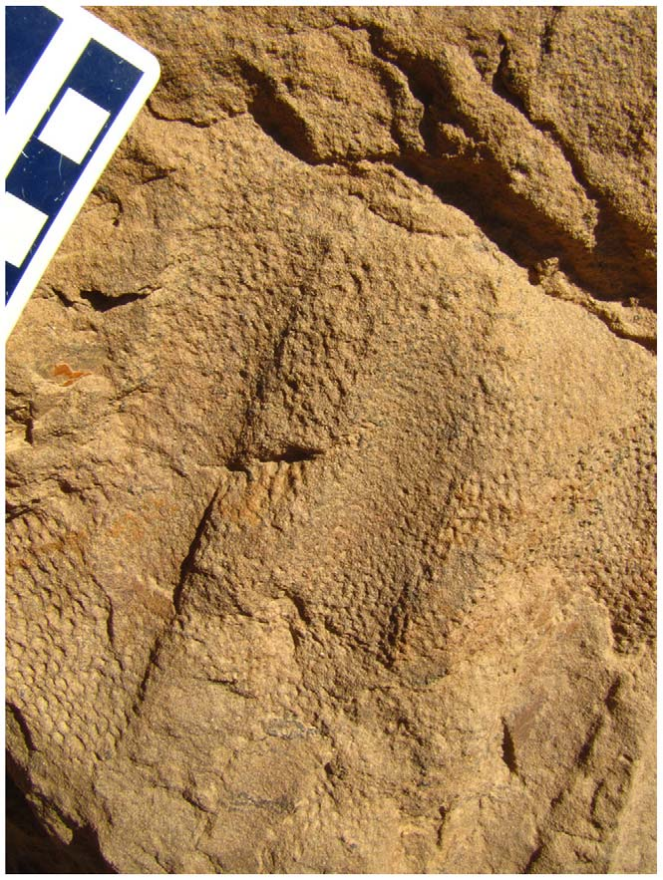
Field photograph of the integument from the flank of a subadult *Saurolophus angustirostris*. Note impressions of the ribs. Scale bar in cm.

Integument from the over the rib cage of *Saurolophus* is not well known and is present only in *S. angustirostris*. Basement-scales in a subadult specimen from the Dragon's Tomb (Fig. 3) appear somewhat distorted. Individual scales are 2–3 mm in diameter and best described as pebbly or irregular in outline; however, their low relief and small size make observations difficult. There does not appear to be any variation in size or morphology. Juveniles (represented by isolated patches of integument on the ribs of ZPAL-MgD-1/159) appear to follow a similar integumentary pattern.

Skin is best represented on the tails of both species of *Saurolophus*. The holotype of *S. osborni* retains skin impressions in two areas. One block measuring 18 cm long contains two fairly complete caudal vertebrae with neural spines. Both centra are 7 cm in length, and probably represent caudal vertebrae six and seven where the tail has been broken on the original specimen ([22]: pl LXIII) presumably to facilitate removal during excavation. Unfortunately, few individual scales are actually distinguishable despite a relatively extensive veneer of integumentary impressions across the specimen. Those that are discernable on the centra are hexagonal (2.5 mm in diameter) and separated from each other by 1 mm wide bands of interstitial tissue. Scales on the neural spines are larger and hexagonal, although they are difficult to discern. One measurable scale at the base of the neural spine is 4 mm in diameter. It is possible that these scales were arranged into cluster areas, although the poor preservation prevents definite identification of these associations.

A second fragment from an unidentified area on the tail of AMNH 5220 measures 14×9 cm. Scales are hexagonal and of variable size. Although no directional data was available, scales measure 6×6 mm, 6×9 mm, 5×4 mm, and 6×7 mm and are arranged haphazardly. At least one incompletely-preserved feature-scale measures ∼25 mm long; its edges are drawn into tapering points to form a multi-pointed star. Each point fits between the edges of two adjacent regular hexagonal scales. When complete, as many as fifteen points probably surrounded the large scale.

**Figure 4:**
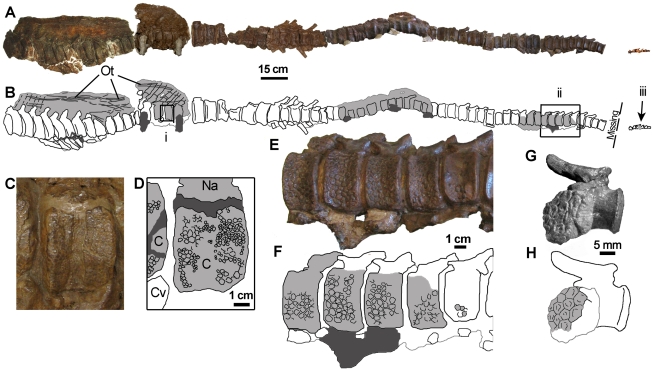
Tail integument of *Saurolophus osborni* (AMNH 5271). (A) Composite photograph and (B) interpretive drawing of caudal series. Note a section of distal vertebrae is missing. *i, ii,* and *iii* identify enlarged images in (C, D), (E, F), and (G, H), respectively. Light grey regions denote areas of skin impressions. Dark grey indicates plaster reconstruction. C, centrum; Cv, chevron; Na, neural arch; Ot, ossified tendons.

The caudal series of AMNH 5271 preserves the most extensive tracts of integument of any specimen of *S. osborni*, spanning the entire length of the tail, albeit discontinuously (Fig. 4). The most proximal vertebrae retain the latticework of ossified tendons along the flanks of the elongate neural spines. Basement-scales on the centra are arranged into cluster areas of small (1–2 mm diameter) and large (4–5 mm) scales (Fig. 4C, D). These patches grade into one another. Individual scales are typically hexagonal, although others are irregular. Scales on the neural spines appear to follow this general pattern, where they are less well preserved. The skin apparently did not extend high above the tips of the neural spines by way of a dorsal ‘frill’, and there is no indication of midline feature-scales. However, it is possible that this area was lost during recovery of the specimen. More distally, the scales are variably hexagonal, pentagonal, or irregular (Fig. 4E, F). Scales are vaguely corrugated and are typically 4–6 mm in diameter. Rare feature scales 6–8 mm long are found close to the chevrons. These differ from the regular basement-scales in that they are irregularly shaped and dorsoventrally taller than they are long.

Scales on the most distal part of the tail are evenly spaced, regular-sided hexagons and pentagons 3 mm in diameter with slightly depressed centres (Fig. 4G, H).

**Figure 5:**
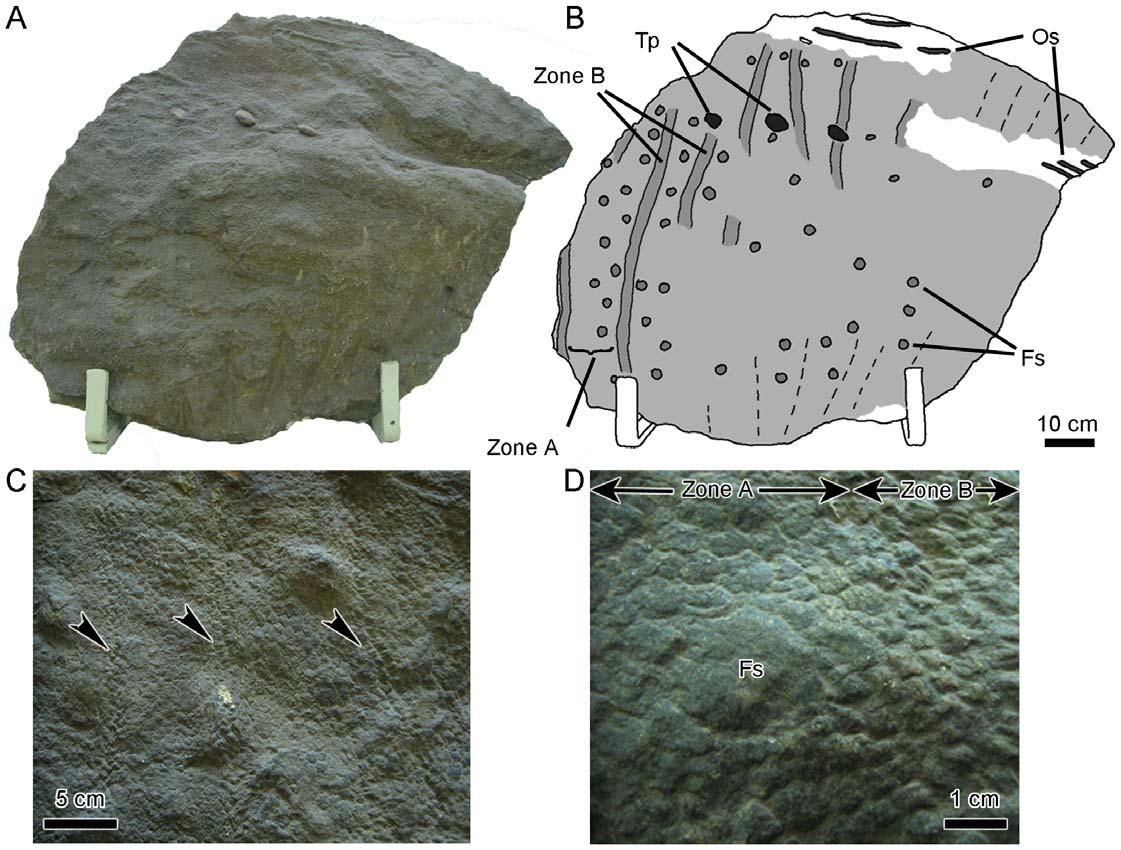
Proximal-most tail integument of adult *S. angustirostris* (PIN 3738). (A) photograph and (B) interpretive illustration. Light grey indicates extent of preserved integument. Dashed lines denote wrinkles in the skin. (C) Close-up showing narrow vertical bands of zone B scales (arrowheads). (D) Detail of transition between polygonal basement-scales in zone A and shell basement-scales of zone B. Fs, feature-scale; Os, ossified tendon; Tp, transverse processes of caudal vertebrae.

**Figure 6:**
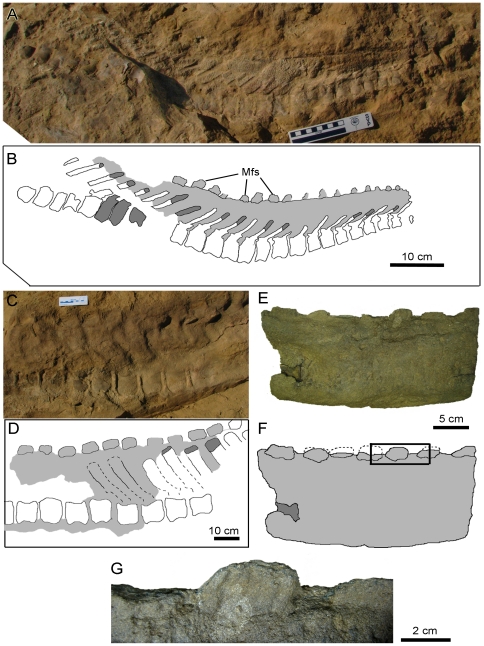
Tail integument of *Saurolophus angustirostris*. (A) field photograph and (B) interpretive illustration of a juvenile caudal series showing distribution of midline feature-scales (Mfs). (C, D) Adult proximal-mid caudal series showing enlarged midline feature-scales. (E, F) Three dimensional skin preservation on mid-distal caudal series of subadult PIN 4216/49. (G) Close-up of midline feature-scales indicated by boxed region in (F). Light grey indicates extent of skin impressions. Dark grey denotes bone. Dashed lines are inferred outlines of structures.

Integument from the tail of *S. angustirostris* varies considerably along its length. Proximally, basement-scales are arranged into alternating, dorsoventrally-oriented bands, herein referred to as zones A and B (PIN 3738, PIN 3747, UALVP 52787; Fig. 5). Zone A bands are approximately 60 mm wide and consist of regular polygonal scales ranging from 4–6 mm in diameter. Slightly larger scales (10 mm) of the same morphology are unevenly interspersed throughout the basement-scales. Other feature-scales are smooth, sometimes multi-pointed, domed shields 25–30 mm in diameter. Shield feature-scales are arranged into a grid-like pattern with individual scales set approximately 4 cm from the neighboring shield feature-scales. Some variation exists in their arrangement, presumably as a result of stretching/wrinkling of the original skin. Zone A grades into zone B, which is immediately recognizable by smaller basement-scales and the absence of feature scales. Zone B bands are comparatively narrow (approximately 20–30 mm wide) and are composed of irregular, corrugated basement-scales 3–4 mm in diameter. Midline feature-scales form a near-continuous series dorsal to the neural spines along the caudal vertebrae (Fig. 6). Individual scales do not correspond perfectly with the tip of each neural spine. Because skin impressions are not known from the cervical region or above the dorsal vertebrae, it is unclear whether or not the midline feature-scales continue along these regions. In the largest individuals (UALVP 52748), midline feature-scales measure up to 80 mm long and 40 mm high (Fig. 6C, D). The lateral surfaces of the midline feature-scales have several dorsoventrally oriented ridges and grooves (Fig. 6G), similar to those described for cf. *Edmontosaurus* (MOR V 007; [5]).

The distal half of the tail in *S. angustirostris* is devoid of distinct dorsoventral bands or zones and lacks feature-scales. Instead, the integument is comprised of an even covering of polygonal basement-scales. Midline feature-scales continue distally and appear to be present almost to the tip of the tail (UALVP 52824).

### Appendicular regions

**Figure 7:**
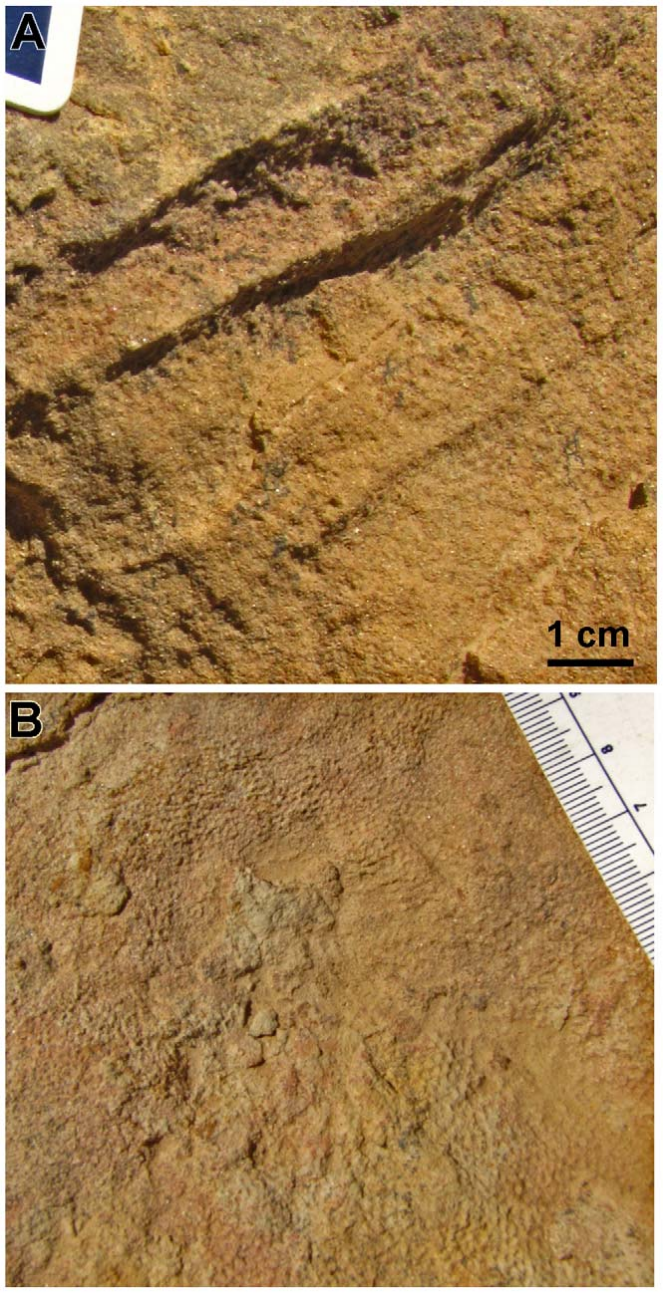
Field photographs of pebbly basement-scales in *Saurolophus angustirostris*. (A) Forearm and (B) shoulder girdle, superficial to the scapula. Longitudinal ridges in A are folds in the integument; distal is to the right.

Although skin is not known from the forelimb of *S. osborni*, the entire forelimb of *S. angustirostris* (UALVP 52781, UALVP 52786) was covered in a uniform arrangement of 1–2 mm wide pebbles even in relatively large individuals (Fig. 7). This pattern persisted over the shoulder joint, humerus, and forearm; however, skin from the manus of *Saurolophus* is unknown. There are apparently no feature-scales, cluster areas or variation within the forelimb.

**Figure 8:**
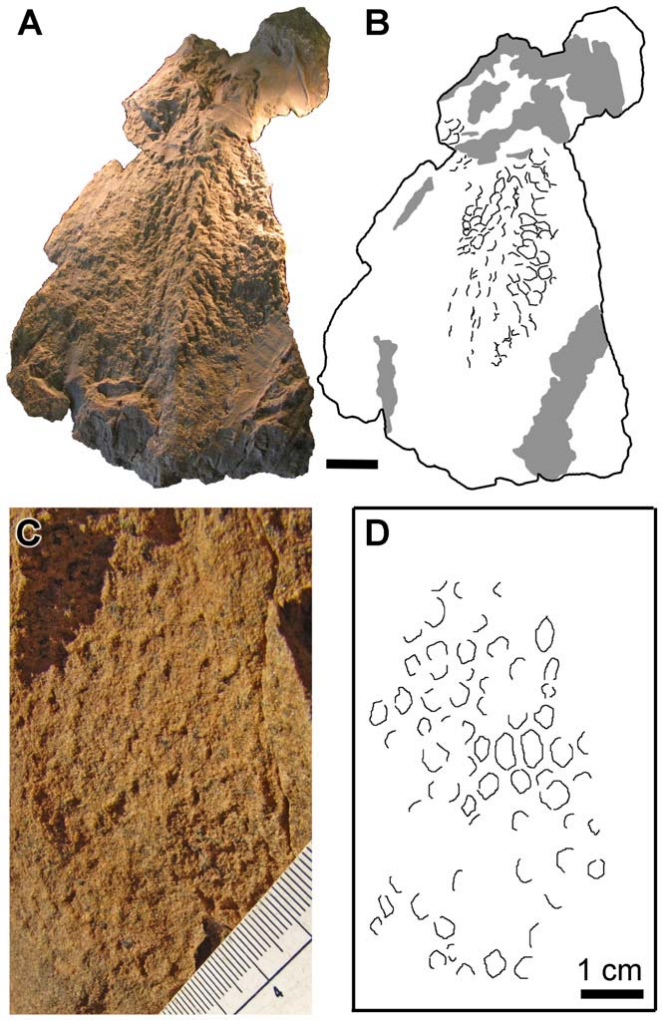
Pelvic integument of *Saurolophus*. (A, B) *S. osborni* (AMNH 5220) from the lateral surface of the iliac body. (C, D) Field photograph of *S. angustirostris* integument preserved between the sacral ribs of a subadult individual. Grey areas denote areas obscured by plaster.

In *S. osborni*, only AMNH 5220 retains skin impressions from the pelvic region. Three fragments from the body of the ilium show a basement of regular hexagonal scales 3×4 mm (Fig. 8A, B). There is no indication of any variation in these scales and no feature-scales were observed. A fragment measuring 10 cm long and 7 cm wide, taken from the shaft of the ischium, preserves a non-uniform arrangement of irregular, multi-pointed basement-scales. The number of points is variable and difficult to discern because of the imperfect preservation. Individual scales are not regularly shaped; scales may be equi-dimensional, constricted at their midpoint, or pinched on one end.

Skin from the pelvic region of *S. angustirostris* is known only from a small patch between the sacral ribs of a subadult individual (Fig. 8C, D). Basement-scales are irregular and measure 5 mm in diameter. Feature-scales and other variations are absent; however, the extent of skin is too small to confirm the presence/absence of variation or feature-scales.

**Figure 9:**
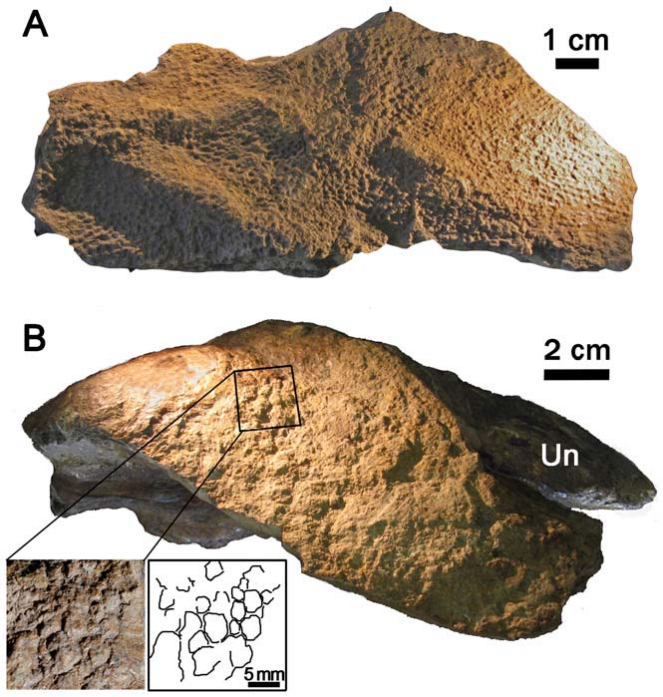
Skin impressions from the left pes of *S. osborni* (AMNH 5220). (A) Skin from the anterior surface of the metatarsus. (B) ?Lateral view of an unidentified pedal digit with the ungual (un) still intact showing irregular scales (inset).

Skin from the hindlimb of *S. osborni* is unknown except for the metatarsus and pes. In the holotype (AMNH 5220), the anterior surface of the left metatarsus was covered in regularly-spaced pebbles approximately 2 mm in diameter. Although most scales are sub-circular in outline, others appear to be hexagonal (Fig. 9A). The pes is represented by a block containing two of the pedal unguals that were not installed as part of the panel mount. The pes was partially disarticulated after death (as seen on the original panel mount), and one ungual is rotated behind the other. Therefore, the exact original position of the skin fragment is unknown, but it is likely from the top of the foot. Skin impressions over the dorsal surface of the digit measure 19×10 cm in total area. The basement-scales are slightly elongated hexagons (6×7 mm). Smaller scales also occur but their positioning is apparently random. A portion of skin that lies adjacent to the ungual is separated from the remainder of the impressions by a shallow depression. Scales on this surface are small, irregular pebbles 2–3 mm in diameter, which may represent a displaced portion of the digital pad or the lateral surface of the digit (Fig. 9B).

The hindlimb integument of *S. angustirostris* is known from both juveniles and subadult specimens. The proximal region of left femur of the juvenile ZPAL-MgD-1/159 preserves three discrete patches of skin. The largest is 140×65 mm on the anterior aspect between the head and the greater trochanter. On the anterolateral edge of the greater trochanter, a second patch adheres closely to the bone and can be traced faintly for most of the preserved length of the element (∼180 mm). A third patch, anterior to the greater trochanter, measures 90×45 mm. In all three areas, basement-scales are corrugated and irregular. Most measure 3–4 mm in greatest dimension but are interspersed with smaller 1 mm wide pebbles. Relief is minimal, although some scales have small keels and other presumed feature-scales (4–5 mm across) are more domed and elevated relative to the surrounding scales. No distributional pattern is evident for these feature-scales.

**Figure 10:**
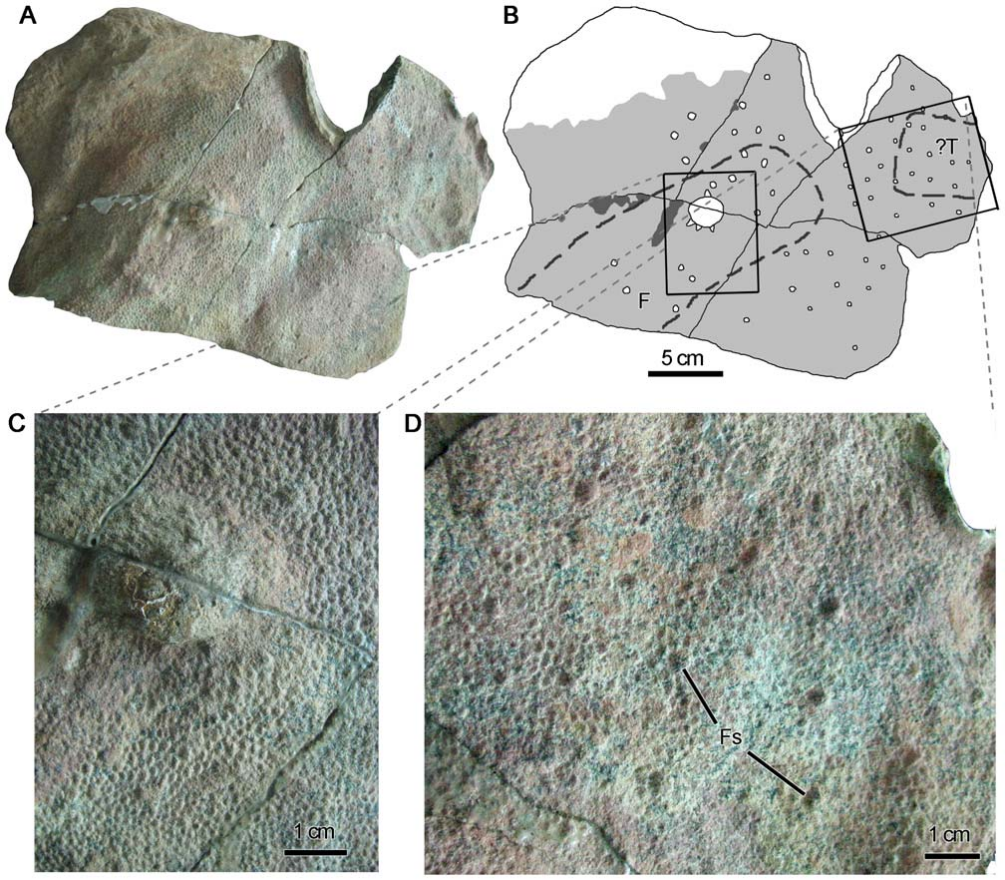
Hind limb skin impressions of *Saurolophus angustirostris*. Right leg of juvenile *S. angustirostris* (ZPAL-MgD-1/159). (A) Photograph and (B) interpretive drawing showing distribution of shield feature-scales (fs). Thick dashed line indicates outline of femur (f) and ?tibia (?t) exposed on the opposite surface of the block. (C) Close-up of large multi-pointed shield feature-scale. (D) Detail of integument.

The distal half of the femur and proximal tibia was dominated by irregular-to-subcircular basement-scales 3 mm in diameter. Arranged at intervals of 2–3 cm are larger feature-scales ∼7 mm across (Fig. 10). These are circular and domed and the periphery of each shield is ornamented by a series of fine, radiating grooves and ridges. The shields are further arranged into rows 20–25 mm apart, and each row is staggered relative to the two adjacent rows. A single multi-pointed shield close to the distal end of the femur is imperfectly preserved but measures 40 mm across (Fig. 10C). This shield feature-scale is strongly domed but is otherwise smooth. The central shield is 20 mm across and is surrounded by at least four triangular points spaced at irregular intervals, but may have been surrounded by as many as eight points.

Skin around the ankle joint and dorsal surface of the pes is comparatively simple, consisting of a basement of uniform pebbles 1–2 mm in diameter devoid of variation or feature-scales (UALVP 52785).

### Miscellaneous (Fig. 11)

**Figure 11:**
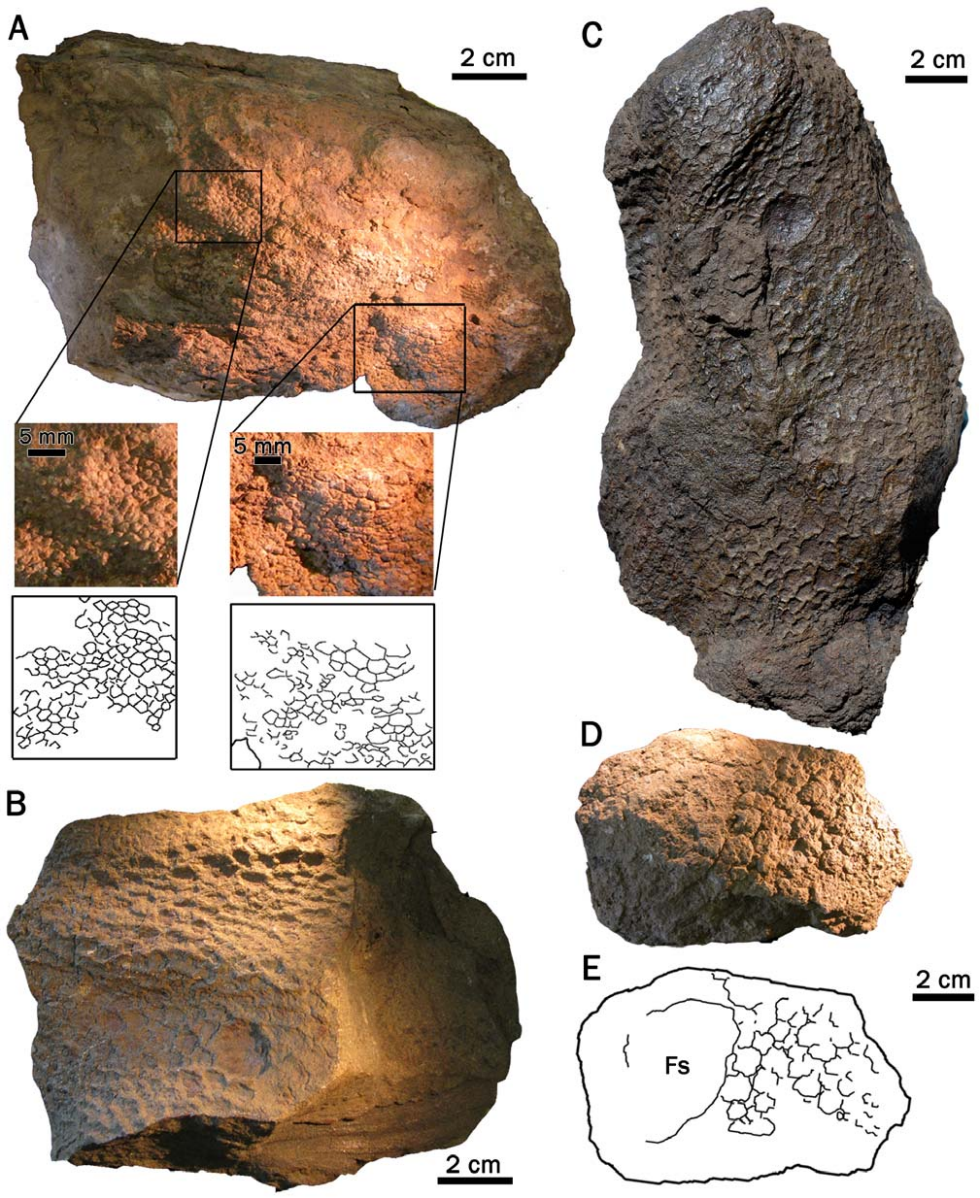
Miscellaneous skin impressions of *Saurolophus osborni*. (A) AMNH 5220; (B) AMNH 5221; (C) AMNH 5221; (D) AMNH 5220. Fs, feature scale.

Several blocks of integument of unknown anatomical origin were found associated with the type and paratype of *S. osborni*. These are described here because they represent otherwise unknown morphologies and/or provide insight into the variation of the integumentary covering of this species.

Three patches of miscellaneous skin from AMNH 5220 were identified. One patch measuring 13×8 cm preserves four-to-six-sided, polygonal basement-scales ranging from 1–3 mm in diameter. Scales are arranged into a mosaic of similar-sized scales, although the specimen is too small to permit identification of the outline of these patches. One patch of large (3 mm) scales is 30 mm in maximum dimension.

Two other blocks measuring 13.5×10 cm and 11.5×7 cm display a basement of regular hexagonal scales 9–11 mm in diameter, which show some degree of imbrication. The posterior, or ‘free’ edges of the scales are ‘stepped’ and show wrinkles perpendicular to the truncated edge. The surfaces of the scales are roughened. Basement-scales in one specimen increase in size until they abut a single shield feature-scale at the edge of the block. The shield measures approximately 35×45 mm; however, some of its edges are damaged, and its full extent cannot be ascertained. Where it is better preserved, its edges occupy the space between neighbouring basement-scales to form a multi-pointed star.

A single miscellaneous block from AMNH 5221 measures 8.5×9 cm. The skin is wrinkled, and the weakly pentagonal or hexagonal basement-scales are variably sized (4×5 mm, 2×3 mm, 6×4 mm), probably owing to the stretching and constriction of the skin. Up to 2 mm of interstitial material separates individual scales. A single preserved shield feature-scale measures 15 mm in diameter. Curiously, it is multi-pointed on one hemisphere whereas the other edge is a relatively smooth (non-pointed) arc.

## Discussion

### Comparison of *S. osborni* and *S. angustirostris*

Comparisons of *S. angustirostris* scale architecture with the lesser-known integument from *S. osborni* provide a test of the taxonomic utility of scale morphology between closely related dinosaur species. Comparisons suggest that these species can be differentiated solely by characters of the skin and therefore support the hypothesis that scale architecture can be used to identify certain hadrosaurid species. This finding is all the more intriguing considering the osteological similarity between the two species of *Saurolophus*. It also appeals for the inclusion of integumentary features (where possible) in the description and potentially the diagnosis of new and existing hadrosaurid taxa.

**Figure 12:**
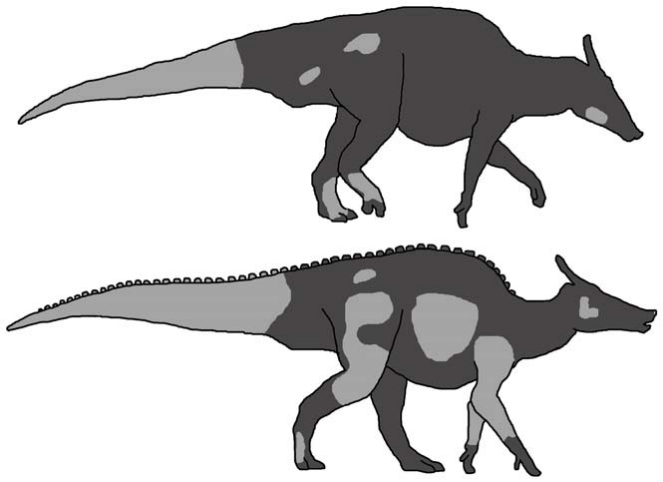
Regions of skin impressions (light grey) currently known for species of *Saurolophus*. (A) *S. osborni* and (B) *S. angustirostris*.

As with earlier studies of hadrosaur skin impressions [16], comparisons are necessarily incomplete as equivalent regions of the integument are uncommon between specimens (Fig. 12). Nevertheless, several comparisons and observations can be made to permit some generalizations about the differences and similarities in the scale architecture between species of *Saurolophus*.

**Figure 13:**
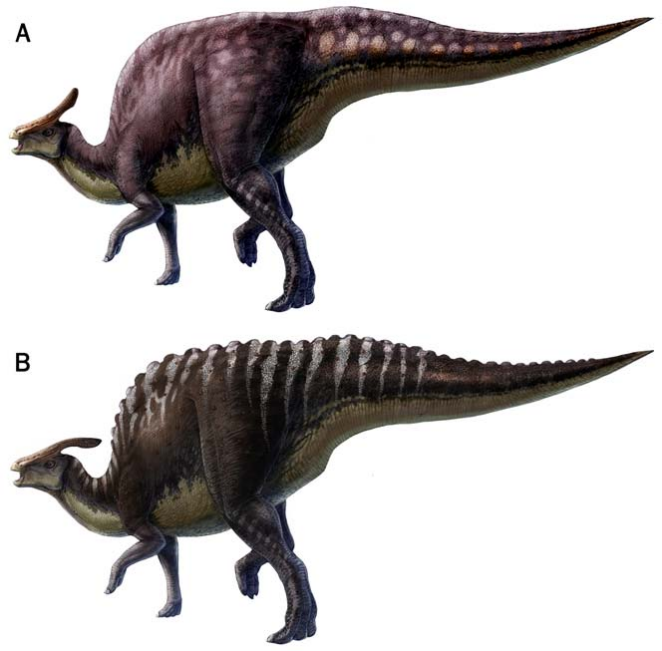
Soft tissue reconstructions of *Saurolophus* based on preserved skin impressions. Variations in scale arrangement and patterning are used here as a basis for possible colour patterns, which are particularly evident in the caudal region. (A) *Saurolophus osborni* showing mottled appearance of tail integument. (B) *Saurolophus angustirostris* showing midline feature-scales and banded pattern on the tail. Illustration by L. Xing and Y. Liu.

The area of most overlap between species of *Saurolophus* is undoubtedly the tail. The presence of cluster areas of basement-scales in *S. osborni* is markedly different to the complex patterning in *S. angustirostris*. The latter species is typified by vertical bands of differentiated basement-scales and shield feature-scales at the base of the tail and a more homogeneous covering of polygonal basement-scales distally (Fig. 13). Moreover, the midline feature-scales observed in both juvenile and adult *S. angustirostris* cannot be confirmed in *S. osborni*; however, this region is less well known in *S. osborni*. The replication of basic scale patterns between specimens (i.e. individuals) of *S. angustirostris* (for example, the banding in the tail regions of PIN 3738, PIN 3747, and UALVP 52787) lends support to the hypothesis that skin impressions are relatively conservative within species of *Saurolophus*. Nevertheless, the addition of new specimens in the future will help build a more complete view of individual and possibly sexual variation.

Multi-pointed shield feature-scales are apparently unique to both species of *Saurolophus* (see following discussion on interspecific variation), having been observed on the hindlimb of *S. angustirostris* and from the tail and unidentified areas of *S. osborni*. At this stage, the multi-pointed shield morphologies are too rare to understand their distribution over the body.

Integumentary patterns in *S. angustirostris* appear to have remained relatively unchanged throughout ontogeny. The presence of domed shield feature-scales from the tail and hindlimb of immature and larger, more mature *S. angustirostris*, suggests at least some consistency in the scale morphology of this taxon through the observed portions of ontogeny. Similarly, midline feature-scales are present on both small and large individuals. Admittedly, the areas of overlap between young and old individuals are limited, so consistent scale architecture between age groups cannot be argued with certainty. In addition, the skin of hatchling and ‘yearling’ *Saurolophus* is as yet unknown. If true, however, the presence of similar markings between young and old individuals may have helped with intra- and inter-species recognition.

### Comparison with other hadrosaur skin impressions

Although a comprehensive comparison with other hadrosaurid skin impressions is beyond the scope of this paper, excellent descriptions of the integument are available for *Edmontosaurus annectens* [2] and *Corythosaurus casuarius* [19]. Additional skin impressions are also known from *Brachylophosaurus canadensis* [27], *Gryposaurus notabilis* ( = *G. incurvimanus*; [28]), *Parasaurolophus walkeri* [16], *Lambeosaurus magnicristatus* [26], *Lambeosaurus lambei* [16] as well as several other unidentified hadrosaurids. Such specimens permit some comparisons and generalizations to be made about hadrosaurid integument.

Skin impressions from the cranium and mandibles are notably rare among hadrosaurids. In addition to those of *Saurolophus*, facial skin impressions have been observed on only two other specimens [2], . Scales preserved on the quadrate and the region immediately posterior to it in *E. annectens* (AMNH 5060) consist of cluster areas of polygonal scales interspersed within a larger area of pebbly basement-scales [2]. Pebbly basement-scales were observed in *S. angustirostris*; however, there is no evidence of cluster areas of morphologically distinct scales. Admittedly, the patches of skin in *S. angustirostris* (ZPAL-MgD-1/159) are too small to conclusively dismiss the presence of cluster areas on the skull of this taxon. Nevertheless, cluster areas were not observed on any other part of the body, so there is little reason to believe they existed on the skull of *S. angustirostris*. Wegweiser et al. [20] described the scales from the anterior part of the dentary of an indeterminate (possibly lambeosaurine) hadrosaur (UW-39449) from the Lance Formation. The scales are polygonal, several millimetres in diameter, and ornamented by fine corrugations that fan out from the ventral margin. Although *S. osborni* also possessed polygonal scales on the dentary, ornamentation such as described on the Lance hadrosaur is absent.

Scales from the shoulder girdle and forelimb of *S. angustirostris* differ from those seen in other hadrosaurids. In *S. angustirostris*, this entire region is populated with a uniform basement of pebbles. In *E. annectens* (AMNH 5060), pebbly basement-scales are present on the ventral surface of the forearm and medial surface of the humerus; however, the humerus also bears small cluster areas of polygonal basement-scales [2]. The dorsal surface of the entire arm in *E. annectens* (AMNH 5060) and the ventral surface of the forearm in *B. canadensis* (JRF 115) were covered in a uniform basement of polygonal scales up to 10 mm in diameter [2], [27]. Undifferentiated polygonal basement-scales are also present on the anterior surface of the humerus of *Lambeosaurus magnicristatus* (TMP 66.4.1; [26]).

Lull and Wright [16] recognized two categories of scale patterns on the flanks. The first is comprised of an undifferentiated covering of polygonal basement-scales (*C. casuarius* (AMNH 5240), *L. lambei* (CMN 8703), *G. notabilis* [ROM 764], *Parasaurolophus walkeri* [ROM 768] and also *S. angustirsotris*). The second form consists of a mosaic of similar-sized scales arranged into cluster areas and is seen only in *E. annectens* (AMNH 5060; [2]). Thoracic integument is unknown from *S. osborni*.

The scale architecture of hadrosaurs is best represented along the tail and demonstrates the morphological diversity of integument in this group. Cluster areas on the proximal caudal region of *S. osborni* are reminiscent of the body covering of *E. annectens* (AMNH 5060), but the multi-pointed and irregular feature-scales from other parts of the tail are unlike previously described hadrosaur integument. Undifferentiated, polygonal basement-scales are found on *C. casuarius* (AMNH 5240) and *L. lambei* (CMN 8703), which is similar to the distal tail of *S. angustirostris*. Proximally, the caudal integument of *S. angustirostris* bears some resemblance to that of an incomplete skeleton from the Dinosaur Park Formation (‘*Trachodon marginatus*’; AMNH 5894 [3]). The tail of ‘*T. marginatus*’ was covered in polygonal basement-scales along with sparse, radially-ornamented shield feature-scales [3]. The vertical banding (as described here for *S. angustirostris*) is absent in ‘*T. marginatus*’ and all other described hadrosaurid specimens. Broad areas of irregular basement-scales from the tail of an indeterminate hadrosaur (NMMNH P-2611) from the Late Campanian of New Mexico show ornate radial ornamentation and include radially-ornamented shield feature-scales [29]. Midline feature-scales as described here for *S. angustirostris* have also been identified on the tail of cf. *Edmontosaurus* (MOR V 007; [5]) and over the dorsal vertebrae of *Brachylophosaurus canadensis* (JRF 115; [27]) and *Gryposaurus notabilis* (ROM 764; [28]).

Pelvic and abdominal skin of *Saurolophus* appears to have been uniformly distributed but differs between species in the contours of the basement-scales. *S. osborni* apparently consisted of regular hexagonal basement-scales along the ilium and irregular basement-scales along the ischium. In its relatively uniform arrangement, pelvic skin of *S. osborni* most closely resembles that of *Gryposaurus notabilis* (CMN 2278) and *Lambeosaurus magnicristatus* (TMP 66.4.1), both of which had an undifferentiated basement of polygonal scales [4], [26]. Only two other taxa preserve skin in this region, both of which differ from *S. osborni*. *E. annectens* (AMNH 5060) retained a mosaic of cluster areas that was present elsewhere on the body [2] and *C. casuarius* (AMNH 5240) possessed a basement of polygonal scales interrupted by shield feature-scales arranged into close-set rows [19]. The single patch of irregular scales associated with *S. angustirostris* differs from the polygonal scales in most other taxa, except *S. osborni* but this patch is too small to permit detailed comparisons.

Aside from the pedes of *C. casuarius* (AMNH 5240), very little is known of the integument from the hind limbs of hadrosaurids. ZPAL-MgD-1/159 (*S. angustirostris*) therefore provides the most complete picture of the hind leg of any hadrosaur described to date. Skin from the front of the proximal femoral region of *Lambeosaurus lambei* ( = *L. clavinitialis*, CMN 8703; [30]) consists of small, polygonal basement-scales devoid of feature scales. This pattern is similar to patches of skin preserved on the fibula of *E. annectens* (AMNH 5060). Skin from the hindlimb of *S. angustirostris* differs markedly from these specimens: the proximal femur consists of irregular, radially-corrugated scales and sporadically-placed shield feature-scales. More distally, the existence of larger shield feature scales over the knee and a large, multi-pointed shield on the distal femur appear unique to this species. The arrangement of polygonal and pebbly scales associated with the pes of both *S. angustirostris* and *S. osborni* is not unlike that observed on *C. casuarius* (AMNH 5240 [1]).

The recognition of morphologically distinct scales and scale patterns on different parts of the body urges for better information-taking of skin impression fossils. The application of a standardized terminology in scale descriptions will be critical in helping to compare hadrosaurid skin impressions and to elucidate further differences and similarity between taxa. It is also necessary for detailed information to be kept when preparing specimens that preserve skin impressions, especially when these impressions are removed from the context of the skeleton. Specifically, a photographic and written record of the orientation and position of the body should accompany skin impression fossils so that appropriate comparisons may be made not only between taxa but between body regions.
